# A Novel Scoring System Predicting Red Blood Cell Transfusion Requirements in Patients Undergoing Invasive Spine Surgery

**DOI:** 10.3390/jcm13040948

**Published:** 2024-02-07

**Authors:** Alina Schenk, Jonas Ende, Jochen Hoch, Erdem Güresir, Josefin Grabert, Mark Coburn, Matthias Schmid, Markus Velten

**Affiliations:** 1 Institute for Medical Biometry, Informatics and Epidemiology, University Hospital Bonn, 53127 Bonn, Germany; schenk@imbie.uni-bonn.de (A.S.); matthias.schmid@ukbonn.de (M.S.); 2 Department of Anesthesiology and Intensive Care Medicine, University Hospital Bonn, 53127 Bonn, Germanyjosefin.grabert@ukbonn.de (J.G.); mark.coburn@ukbonn.de (M.C.); 3 Institute for Experimental Hematology and Transfusion Medicine, University Hospital Bonn, 53127 Bonn, Germany; 4 Department of Neurosurgery, University Hospital Bonn, 53127 Bonn, Germany; erdem.gueresir@medizin.uni-leipzig.de; 5 Department of Neurosurgery, University Hospital Leipzig, 04103 Leipzig, Germany; 6 Department of Anesthesiology and Pain Management, The University of Texas Southwestern Medical Center, Dallas, TX 75390, USA

**Keywords:** blood bank, storage, blood shortage

## Abstract

**Background:** Access to blood products is crucial for patient safety during the perioperative course. However, reduced donations and seasonally occurring blood shortages pose a significant challenge to the healthcare system, with surgeries being postponed. The German Blood Transfusion act requires that RBC packages become assigned to an individual patient, resulting in a significant reduction in the available blood products, further aggravating shortages. We aimed to develop a scoring system predicting transfusion probability in patients undergoing spine surgery to reduce assignment and, thus, increase the availability of blood products. **Methods:** The medical records of 252 patients who underwent spine surgery were evaluated and 18 potential predictors for RBC transfusion were tested to construct a logistic-regression-based predictive scoring system for blood transfusion in patients undergoing spine surgery. **Results:** The variables found to be the most important included the type of surgery, vertebral body replacement, number of stages, and pre-operative Hb concentration, indicating that surgical specification and the extent of the surgical procedure were more influential than the pre-existing patient condition and medication. **Conclusions:** Our model showed a good discrimination ability with an average AUC [min, max] of 0.87 [0.6, 0.97] and internal validation with a similar AUC of 0.84 [0.66, 0.97]. In summary, we developed a scoring system to forecast patients’ perioperative transfusion needs when undergoing spine surgery using pre-operative predictors, potentially reducing the need for RBC allocation and, thus, resulting in an increased availability of this valuable resource.

## 1. Introduction

Invasive surgical procedures require access to various blood products in the case of severe bleeding and consecutive transfusion requirements [1]. Blood products have been acknowledged as a critical recourse in healthcare and, accordingly, the WHO has recommended strategies to ensure blood product supply meets the demand, clinical guidelines on the appropriate use of blood products, and strategies to reduce transfusion rates [2]. Although the implementation of patient blood management (PBM) programs utilizing multiple strategies have significantly reduced transfusion rates, pre-operative anemia has a high prevalence in surgical patients and blood products are still required for dedicated procedures, associated with a likely transfusion risk [3,4]. Furthermore, transfusion thresholds remain to be determined and are being investigated for individual patients and specific procedures [5,6].

In Germany, the transfusion of blood and blood products is regulated by the German Medical Products Act (Arzneimittelgesetz) and the German Blood Transfusion Act (Transfusionsgesetz), setting standards for donor screening, blood testing, product storage, and transfusion administration to ensure the safety of patients receiving transfusions [1]. In addition to worldwide established tests for various infectious diseases, such as HIV, syphilis, hepatitis B and C, as well as AB0 compatibility, each unit of red blood cells (RBCs) has to be tested for additional individual markers to the recipient, including the Rh factor and further rare antigens, to prevent potential transfusion reactions. Also, the German Blood Transfusion act requires that, after testing, the RBC package becomes assigned to an individual patient until it is transfused, the validation testing expires after three days, or the RBC package is cleared from the case. These assignments, required by federal law, result in a significant reduction in product availability and the need for a higher volume of blood donations compared to other countries.

Blood donations are essential to provide adequate amounts of RBC packages. However, despite the importance of blood donations, there has been a trend of reduced blood donations in Germany over the past decade, from 58 to 41 packages per 1000 inhabitants annual. The decreasing availability and shortages of blood products occurs seasonally and poses a significant challenge to the healthcare system, as scheduled surgeries, potentially requiring blood transfusions, need to be postponed due to limited blood products [7].

Invasive spine surgeries, including open fusion and corpectomy, are complex procedures, potentially resulting in significant blood loss, leading to the need for blood transfusion to replace lost blood and prevent serious complications resulting from anemia, including tissue hypoxia, and organ dysfunction. In the context of the current blood product shortage, the pre-operative prediction of the need for RBC transfusion in patients undergoing spine surgery is crucial to reduce the amount of RBC packages assigned to a specific patient during the perioperative course and, therefore, not available for other patients. Reducing the number of assigned RBCs will increase the number of available products and subsequently prevent the postponing of scheduled surgeries.

The aim of the present study was to derive an easy-to-use clinical work and memorable risk score that is pre-operatively applicable to predict the requirement of RBC packages during spine surgery and the subsequent post-operative course.

## 2. Materials and Methods

### 2.1. Source of Data

In accordance with the Declaration of Helsinki, §15 of the Medical Association Nordrheins’ professional code of conduct, and after approval by the ethics committee of the University Medical Center Bonn, Bonn, Germany, (no. 082/13; 009/20), we performed a retrospective analysis of 252 patients who underwent invasive open spine surgery, including corporal fusion and corpectomy, between January and August 2017. Data from the institutional electronical patient data system were included in the present study and the entire perioperative course was analyzed using a retrospective framework. Minimal invasive procedures utilizing advanced technology and specialized techniques, including microdiscectomy, laminectomy, and vertebroplasty, as well as endoscopic approaches, were not included in the present study.

### 2.2. Outcome

As the outcome of interest, we considered the transfusion of one or more RBC packages during the surgery and post-operative course (binary outcome, yes/no). The observational period was terminated after 30 days when the hospital length of stay extended this duration.

### 2.3. Predictors

For score development, we considered, in total, 18 pre-operative covariates comprising patient-specific characteristics (sex, age (years), height (cm), weight (kg), and ASA classification (1, 2, ≥3), premedication (anticoagulant yes/no), the number of prior surgeries (0, 1, and 2)), pre-operative laboratory measures (Hb (g/dL), quick (%), INR, PTT (s), and thrombocytes), and details on the planned surgery (fracture, (yes/no), tumor (yes/no), location (cervical spine, thoracic spine, lumbar spine or combinations thereof), incision (dorsal, ventral), vertebral body replacement (yes/no), and stages (1, 2, 3, and >3)). Continuous variables are reported as mean (SD) and median [min, max]. Categorical variables are reported as absolute and relative frequencies.

### 2.4. Score Development

**Figure 1:**
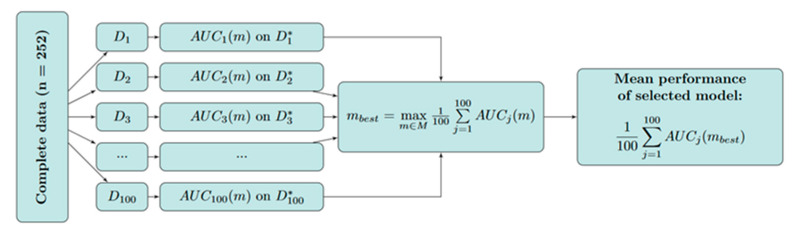
Resampling approach for identifying the best-performing logistic model with respect to the AUC (training cohorts are denoted by $D_{j},\;j=1,\;\ldots ,\;100$; test cohorts are denoted by $D_{j}^{*},\;j=1,\;\ldots ,\;100$).

The probability of transfusion of one or more RBC packages was modeled using logistic regression. For simplicity, but also for statistical reasons (not more than 10 events per covariate), the maximum number of covariates included in our final prediction model was restricted to four [8]. Thus, we considered, in total, 4048 different possible logistic regression models consisting of different combinations of a maximum of 4 out of 18 possible predictors (including the “null model” not containing any covariate). The total number of 4048 models corresponded to the number of possibilities to choose k=0, …, 4 out of 18 available predictors without replacement and disregarding the order of the predictors. In the following, the set of the possible models with the size 4048 is denoted by M and one model out of this set is denoted by m. The best-performing logistic regression model was found by a (nested) resampling algorithm based on the maximization of the area under the curve (AUC; see below and Figure 1). More specifically, our approach comprised the following steps (see Figure 1):

(i) The random division of the complete cohort (*n* = 252) into 100 training cohorts from $D_{1}$ to $D_{100}$ (each containing 80% of all observations, stratified by event/non-event) and 100 test cohorts from $D_{1}^{*}$ to $D_{100}^{*}$ (each containing the remaining 20% of observations) using random sampling.
(ii) Each possible model m∈M was fitted to each training cohort $D_{j},\;j=1,\;\ldots ,\;100$ and the AUC was calculated for each test cohort $D_{j}^{*},\;j=1,\;\ldots ,\;100$, denoted by $AUC_{j}\left(m\right)$.
(iii) The best-performing model was derived by maximizing the mean AUC across all test cohorts.

**Figure 2:**
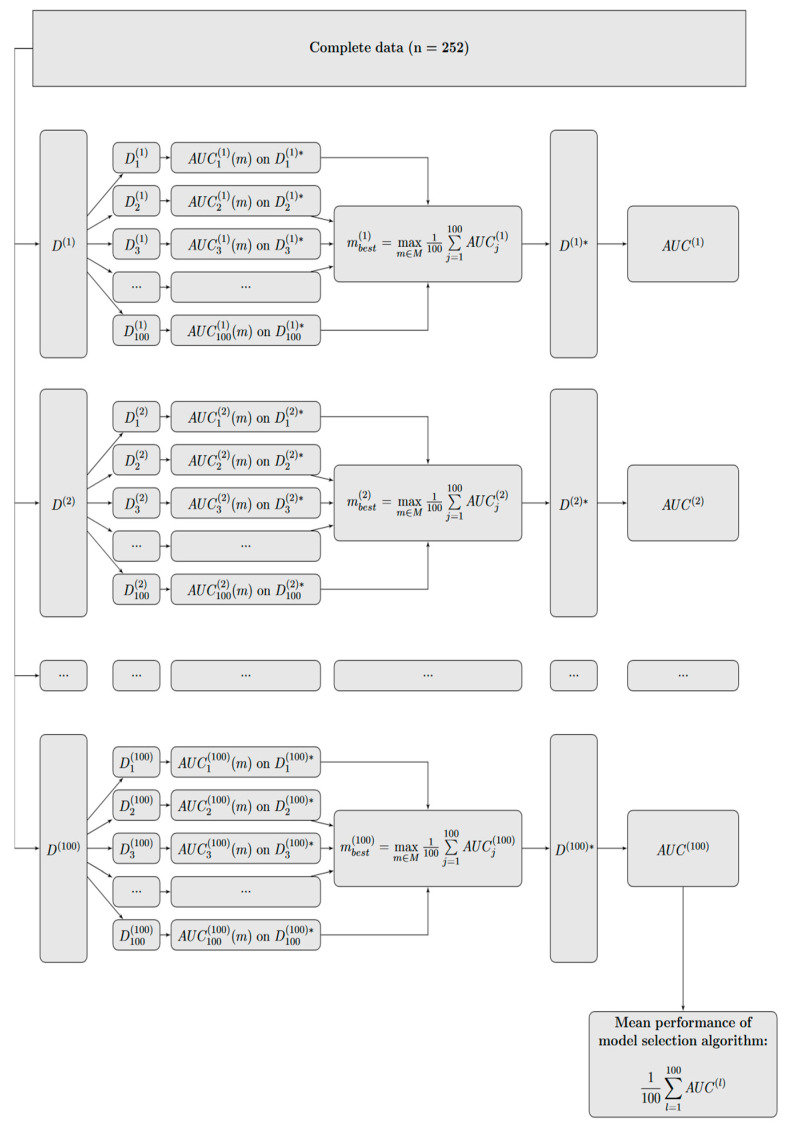
Nested resampling approach for evaluating the performance of the model selection algorithm (derivation cohorts are denoted by $D^{\left(k\right)},\;k=1,\;\ldots ,\;100$; validation cohorts are denoted by $D^{\left(k\right)*},\;k=1,\;\ldots ,\;100$).

The mean performance of the selected model is expressed by the mean AUC across all test cohorts $D_{j}^{*},\;j=1,\;\ldots ,\;100$. For additional validation and to quantify the performance of the model selection algorithm, we applied the resampling approach described above in a nested manner (see Figure 2) [9]. For this purpose, we randomly divided the complete cohort into 100 derivation cohorts (denoted by$\;D^{\left(k\right)},\;k=1,\;\ldots ,\;100$, each containing 80% of the data) and applied the resampling approach described in Figure 1 to each of the 100 derivation cohorts. For each k=1, …, 100, the best-performing model was selected by maximizing the mean AUC across all 100 validation cohorts (denoted by $D_{j}^{\left(k\right)*}$ in Figure 2, each containing 20% of the data) and the mean AUC of the best-performing model on the validation cohort $D_{j}^{\left(k\right)*}$, denoted by $AUC^{\left(k\right)},\;$was calculated. The mean [min, max] of the $AUC^{\left(k\right)}$ was used as the performance measure of the model selection algorithm.

The logistic regression model that was selected as the best-performing model according to the algorithm described in Figure 1 was subsequently applied to the complete cohort (*n* = 252). In the next step, the coefficient estimates of the resulting model fit were used to develop a scoring system assigning risk points to each category of the included risk factors (predictors) and summing them up to obtain a total score (11). Based on this system, we constructed a look-up table allowing for the easy extraction of the corresponding estimated transfusion probabilities. All calculations were performed using the R language and environment for statistical computing (Version 4.1.2). An implementation of the model selection algorithm is showcased in the R-Code in the Supplementary Material XY.

### 2.5. Handling of Missing Data

We conducted a complete case analysis for each considered model m∈M.

## 3. Results

### 3.1. Participants

In total, 61/252 (24.2%) patients received a transfusion of one or more RBC packages during the perioperative course. The transfused patients were, on average, older than the patients who did not receive RBC packages (67.3 (17.6) years vs. 61.0 (17.0) years; see Table 1). The majority of the patients who received a transfusion were classified as ASA ≥ 3 (45.9%), whereas the majority of patients who did not receive a transfusion were classified as ASA = 2 (58.1%; see Table 1). Furthermore, the administration of anticoagulant drugs (including either ASS, Plavix, NOAK, Marcumar, or Heparin) prior to the surgery was more frequently observed in patients who received RBC packages than in patients who did not receive RBS packages (41.0% vs. 26.7%; see Table 1). In total, 214/252 (84.9%) patients had no prior spine surgery at our institution (Table 1). Additionally, 34 patients had a second spine surgery at our institution (i.e., those patients had one prior surgery in our data), and in 4 patients, a third spine surgery was performed (i.e., those patients had two prior spine surgeries in our data). Thus, 38 patients were included at least twice in our analysis, every time with a different surgery. We accounted for this by including the number of prior spine surgeries as a possible covariate in the logistic regression models (see Section 3.2). Note that the pre-operative laboratory measures as well as the details on the planned surgery differed among repeated surgeries.

The patients who received RBC packages exhibited lower pre-operative Hb concentrations (on average, 10.6 (2.14) g/dL), while patients who did not receive RBC packages had, on average, higher pre-operative Hb concentrations (13.3 (1.96) g/dL). Furthermore, the average quick was lower in transfused patients (96.1 (13.5) %) compared to patients who did not require a RBC transfusion (103.0 (14.7) %). Additional pre-operative laboratory measures can be found in Table 2.

A fracture was present in 37.7% of patients who required a RBC transfusion and in 26.2% of patients without a RBC transfusion. Furthermore, a tumor was present in 21.3% of patients with a RBC transfusion and in 15.2% of patients without a RBC transfusion (Table 3). Most of the spine surgeries in both groups were performed from a dorsal approach (88.5% in patients with transfusions and 75.9% in patients without transfusion; Table 3). In patients who received a RBC, the frequency of vertebral body replacement was higher than in patients who did not receive RBC packages (21.3% vs. 8.4%; Table 3). For 48.7% of patients who did not receive RBC packages, the number of stages in the spine surgery was 0, and the frequency of zero stages was only 18.0% in patients who received RBC packages. For more than three-fourths (75.4%, Table 3) of patients who received a transfusion, the number of stages in the spine surgery was greater than 1. The distribution of the type of the planned surgery can be found in Table 3. The surgery was considered as being combined if the planned surgery comprised a combination of cervical spine, thoracic spine, or lumbar spine surgeries.

### 3.2. Prediction Model

The model detected as the best model according to our model selection algorithm described in Figure 1 included the four covariates: type of surgery, vertebral body replacement, stages, and Hb. The best model (evaluated according to Figure 1) showed a good discrimination ability with an average AUC [min, max] of 0.87 [0.6, 0.97]. The performance of the complete model selection algorithm, measured by the average AUC [min, max] in internal validation (evaluated according to the nested resampling approach in Figure 2), was comparably good, with 0.84 [0.66, 0.97].

The coefficient estimates of the logistic model containing the four covariates applied on the complete cohort were used to derive a scoring system according to Sullivan et al. [10]. The reference categories for each risk factor were chosen according to the strength of the risk association, assigning zero points to the groups with the lowest risk and a higher numbers of points to groups with a higher risk (see Table 4; for further technical details, see [10]). Thus, an increase in the score is related to an increased estimated probability of requiring RBC packages during the perioperative course. The continuous covariate Hb was organized into four meaningful categories (<8, [8;12), [12;16], and >16) for assigning risk points in the respective categories. Following Sullivan et al., a constant B reflecting the number of regression units corresponding to one risk point needs to be chosen [10]. For our purpose, we set B as equal to the regression coefficient of the vertebral body replacement, as estimated from the final logistic model. Thus, the constant reflects the increase in RBC transfusion probability associated with the implantation of a vertebral body replacement. The coefficient estimates of the logistic model (a complete cohort with 251 observations) as well as the associated risk points are presented in Table 4. Using the data in Table 4, the individual patients’ transfusion risk can be calculated by summing up all points belonging to the values of the patient’s risk factors. The respective estimated probability of RBC requirement can be extracted from the “look-up” Table 5. Score values can range from 0 (corresponding to a low risk of 0.13%) to 13 (corresponding to a high risk of 98.59%). Two examples on how to use the scoring system are provided in Table 6. For patient 1, a lumbar spine surgery (+1) with a vertebral body replacement (+1) and zero stages (+0) is planned. The pre-operative Hb is 9.1 g/dL (+5). The total score for patient 1 is 1 + 1 + 0 + 5 = 7, corresponding to an estimated probability of RBC package transfusion of 31.13%. For patient 2, a combined surgery (+2) with no vertebral body replacement (+0) and more than three stages (+3) is planned. The pre-operative Hb is 11.7 g/dL (+5). The total score for patient 2 is 2 + 0 + 3 + 5 = 10, corresponding to an estimated probability of RBC package transfusion of 84.91%.

**Figure 3:**
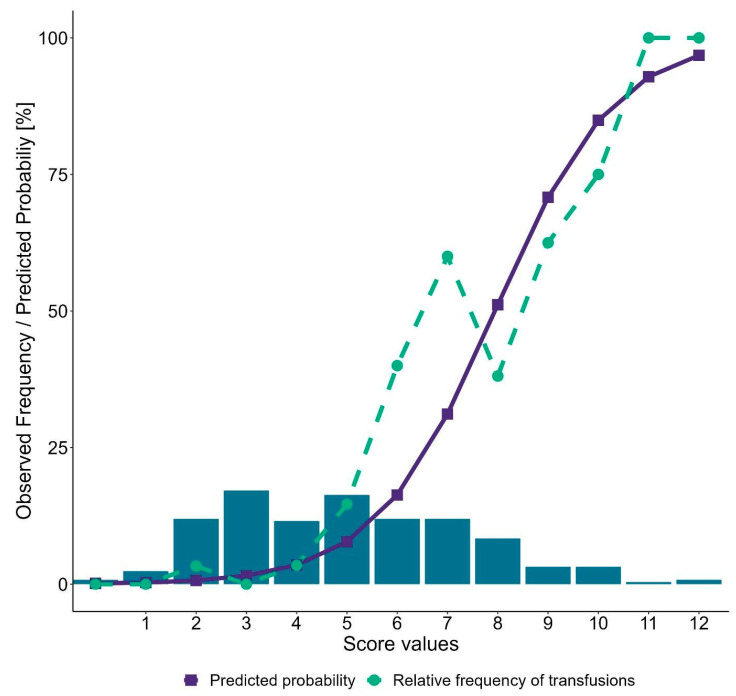
Relative frequencies of score values in the complete cohort (*n* = 251) (green bars) and estimated probability of RBC transfusions (blue line). The dashed green line represents the observed frequency of RBC transfusions in the subgroups of patients reaching the respective score according to the scoring system.

As seen in Figure 3, the total score values in the full cohort ranged between 0 and 12, with the majority of patients having scores between 2 and 8. In general, the observed frequency of RBC package transfusions in the cohort and the probability of requiring transfusions estimated by the scoring system match quite well, indicating that the scoring system is able to accurately discriminate between high- and low-risk patients.

## 4. Discussion

The present study developed a scoring system to forecast perioperative RBC requirements for individual patients undergoing various surgical spine procedures, potentially reducing allocations and, therefore, increasing the availability of blood products in medical systems where individual RBC packages have to be assigned to a specific patient.

Blood product availability is crucial to assure patient safety during surgical procedures. Predicting transfusion requirements in the perioperative course will potentially enable healthcare facilities and providers to improve supply chain management of allogeneic blood, ensuring that blood products are utilized efficiently and cost-effectively [11], but may also evaluate the risk profile of an individual patient to undergo transfusion [12]. However, in the perioperative setting, various factors influence the likelihood of RBC transfusion, including the patient’s condition and surgical procedure. Combining these factors prior to the surgical procedure may help to predict the necessity for transfusion during the perioperative course.

Blood shortages have forced, at least in part due to the development of scoring systems predicting transfusion rates and, thus, red blood cell requirements in order to optimize aliquoting, this valuable resource in perioperative medicine to maintain the performance of planned interventions. Scoring systems have been developed for patients undergoing specific procedures, most notably cardiac procedures or orthopedic joint replacement surgeries [13,14,15,16,17]. Pre-operative lab parameters, including hemoglobin and creatinine, in addition to baseline patients’ characteristics, such as gender, age, height, and weight, as well as pre-existing morbidities or conditions have commonly been used for score development. In cardiac surgery, the EuroSCORE not only predicts survival but is also an established parameter included in the prediction of the perioperative transfusion risk. The ACTA-PORT score analyzed 20,036 patients who underwent cardiac surgeries to develop a model to forecast perioperative RBC transfusion with an area under the curve value of 0.835 (95% CI, 0.810–0.859). Although spine surgeries are frequently associated with blood loss, resulting in a transfusion requirement, no specific score has been developed to date [17].

Our study constructed a simple scoring system forecasting the number of RBC packages required for patients undergoing different spine surgeries by considering, in total, 18 independent pre-operative predictors, comprising the combination of patients’ individual characteristics, laboratory measures, and details on the planned procedure. The variables found to be the most important in our model included the type of surgery, vertebral body replacement, the number of stages, and pre-operative Hb concentration, indicating that surgical specification and the extent of the surgical procedure are more influential than the pre-existing patient condition and medication. Our scoring system demonstrated that an easy-to-use score solely based on four pre-operative characteristics can be a powerful tool with a good discrimination ability and an average AUC [min, max] of 0.87 [0.6, 0.97] in forecasting the transfusion requirements in patients undergoing spine surgery that is even above the AUC rate of the ACTA-PORTS score [17]. Particularly, in our internal validation analysis, the scoring system showed a good discrimination ability and was able to stratify patients at low and high risks of RBC transfusion.

Nie and colleagues developed a score by reviewing the medical records of 565 patients who underwent posterior lumbar surgery and combined these with records from 586 patients who underwent trauma surgery for a femoral fracture. The patients’ clinical characteristics were subjected to multivariate regression analysis and a non-linear regression was performed to predict the probability of intraoperative blood transfusion and the volume of blood used for patients with different scores. However, only octogenarians over 65 years of age were included to develop their model. Furthermore, while, in the case of hip and spine surgeries, different procedures were analyzed, just one surgical approach per procedure was taken into account for model development. In contrast to this, all ages, different anatomical regions, and surgical approaches were included in the development of our model. Furthermore, while Nie and colleagues just focused on patients’ characteristics, independent of surgical approach or the severity of the disease, our model took into account both the severity of the approach and surgical planning.

Regarding the development of our proposed scoring system, there are some limitations to consider. Although we included the impact of the anatomical region (cervical, thoracic, and lumbar) as well as the number of stages in our model, we were not able to analyze more detailed information regarding the specific vertebra or the extent of the surgical approach due to the number of evaluated observations. These details need to be investigated in future prospective studies. Furthermore, as the data were collected retrospectively, the number of included individuals was limited. In this respect, although we performed an in-depth internal assessment of the performance of different models, we highlight the need for future prospective studies that include a larger number of patients for the external validation of the developed scoring system using independent data. Additionally, as the underlying data include patients with spine surgeries only, the scoring system is of course limited to the application to this type of surgeries. However, all different areas of the spine as well as surgical approaches were included. Furthermore, the restriction of including a maximum of four covariates and the simplification of the logistic model to a scoring system might slightly decrease the predictive performance of our proposed scoring system compared to, for instance, machine learning techniques (such as random forests, deep neural networks, or gradient boosting) that might have further improved the predictive performance of our scoring system [18]. However, as we aimed to derive an easy-to-use and understandable scoring system, we did not intend to apply machine learning techniques.

In summary, transfusion prediction in perioperative medicine is essential for ensuring patient safety, optimizing resource management, reducing costs, improving outcomes, facilitating surgical planning, and addressing ethical considerations. By effectively predicting transfusion needs, healthcare providers can personalize patient care, minimize risks, and make an efficient use of valuable blood products, ultimately leading to improved surgical outcomes and improved overall healthcare delivery. The presented scoring system is able to accurately predict the likelihood of patients requiring one or more RBC packages during the surgery as well as the post-operative course and, therefore, addresses all perioperative factors that need to be considered.

## 5. Conclusions

In conclusion, in the current study, we proposed a new scoring system to forecast patients’ perioperative transfusion needs when undergoing spine surgery using pre-operative predictors, potentially reducing the need for RCB allocation and resulting in an increased availability of this valuable resource.

## Figures and Tables

**Table 1:** Patient characteristics.

Patient Characteristics			
	No RBC Package Received (N = 191)	One or More RBC Packages Received (N = 61)	Overall (N = 252)
**Sex**			
Male	96 (50.3%)	33 (54.1%)	129 (51.2%)
Female	95 (49.7%)	28 (45.9%)	123 (48.8%)
**Age**			
Mean (SD)	61.0 (17.0)	67.3 (17.6)	62.6 (17.3)
Median [Min, Max]	65.0 [14.0, 89.0]	69.0 [22.0, 89.0]	66.5 [14.0, 89.0]
**Height**			
Mean (SD)	172 (9.76)	172 (11.3)	172 (10.1)
Median [Min, Max]	170 [150, 198]	172 [140, 196]	172 [140, 198]
Missing	5 (2.6%)	2 (3.3%)	7 (2.8%)
**Weight**			
Mean (SD)	80.3 (17.6)	78.6 (20.6)	79.9 (18.3)
Median [Min, Max]	78.0 [43.0, 150]	76.0 [38.0, 140]	77.5 [38.0, 150]
Missing	2 (1.0%)	0 (0%)	2 (0.8%)
**ASA score**			
1	19 (9.9%)	4 (6.6%)	23 (9.1%)
2	111 (58.1%)	20 (32.8%)	131 (52.0%)
≥3	44 (23.0%)	28 (45.9%)	72 (28.6%)
Missing	17 (8.9%)	9 (14.8%)	26 (10.3%)
**Premedication** **(anticoagulant)**			
No	140 (73.3%)	36 (59.0%)	176 (69.8%)
Yes	51 (26.7%)	25 (41.0%)	76 (30.2%)
**Number of prior** **spine surgeries**			
No prior surgery	171 (89.5%)	43 (70.5%)	214 (84.9%)
One prior surgery	19 (9.9%)	15 (24.6%)	34 (13.5%)
Two prior surgeries	1 (0.5%)	3 (4.9%)	4 (1.6%)

**Table 2:** Pre-operative laboratory measures. The American Society of Anesthesiologists (ASA) physical status score.

Pre-Operative Laboratory Measures			
	No RBC Package Received (N = 191)	One or More RBC Packages Received (N = 61)	Overall (N = 252)
**Hb (g/dL)**			
Mean (SD)	13.3 (1.96)	10.6 (2.14)	12.7 (2.32)
Median [Min, Max]	13.4 [7.70, 19.1]	10.3 [7.00, 15.9]	12.9 [7.00, 19.1]
**Quick**			
Mean (SD)	103 (14.7)	96.1 (13.5)	101 (14.7)
Median [Min, Max]	105 [21.0, 130]	97.0 [63.0, 126]	103 [21.0, 130]
Missing	1 (0.5%)	0 (0%)	1 (0.4%)
**INR**			
Mean (SD)	0.996 (0.161)	1.02 (0.0809)	1.00 (0.146)
Median [Min, Max]	1.00 [0.800, 2.80]	1.00 [0.900, 1.20]	1.00 [0.800, 2.80]
Missing	1 (0.5%)	0 (0%)	1 (0.4%)
**PTT**			
Mean (SD)	24.8 (3.69)	26.1 (6.74)	25.1 (4.62)
Median [Min, Max]	24.0 [18.0, 53.0]	25.0 [18.0, 65.0]	24.0 [18.0, 65.0]
Missing	1 (0.5%)	2 (3.3%)	3 (1.2%)
**Thrombocytes**			
Mean (SD)	278 (104)	291 (105)	281 (104)
Median [Min, Max]	261 [84.0, 792]	275 [94.0, 623]	265 [84.0, 792]
Missing	2 (1.0%)	2 (3.3%)	4 (1.6%)

**Table 3:** Details of the planned surgery.

Details of the Planned Surgery			
	No RBC Package Received (N = 191)	One or More RBC Packages Received (N = 61)	Overall (N = 252)
**Fracture**			
no	141 (73.8%)	38 (62.3%)	179 (71.0%)
yes	50 (26.2%)	23 (37.7%)	73 (29.0%)
**Tumor**			
no	162 (84.8%)	48 (78.7%)	210 (83.3%)
yes	29 (15.2%)	13 (21.3%)	42 (16.7%)
**Type of surgery**			
cervical spine	59 (30.9%)	12 (19.7%)	71 (28.2%)
thoracic spine	25 (13.1%)	7 (11.5%)	32 (12.7%)
lumbar spine	82 (42.9%)	23 (37.7%)	105 (41.7%)
combination	25 (13.1%)	19 (31.1%)	44 (17.5%)
**Incision**			
dorsal incision	145 (75.9%)	54 (88.5%)	199 (79.0%)
ventral incision	46 (24.1%)	7 (11.5%)	53 (21.0%)
**Vertebral body** **replacement**			
no vertebral body replacement	175 (91.6%)	47 (77.0%)	222 (88.1%)
vertebral body replacement	16 (8.4%)	13 (21.3%)	29 (11.5%)
missing	0 (0%)	1 (1.6%)	1 (0.4%)
**Stages**			
0	93 (48.7%)	11 (18.0%)	104 (41.3%)
1	28 (14.7%)	4 (6.6%)	32 (12.7%)
2	38 (19.9%)	19 (31.1%)	57 (22.6%)
3	14 (7.3%)	10 (16.4%)	24 (9.5%)
>3	18 (9.4%)	17 (27.9%)	35 (13.9%)

**Table 4:** Scoring system with coefficient estimates from the logistic model (complete cohort with 251 observations) and associated risk points.

Risk Factor	Coefficient Estimate	Risk Points
**Type of surgery**		
cervical spine	-	0
thoracic spine	0.267	0
lumbar spine	1.099	1
combination	1.783	2
**Vertebral body replacement**		
no	-	0
yes	0.841	1
**Stages**		
0	-	0
1	0.593	1
2	1.805	2
3	2.260	3
>3	2.122	3
**Hb (g/dL)**		
<8	−0.665	7
[8;12)	−0.665	5
[12;16]	−0.665	2
>16	−0.665	0

**Table 5:** Look-up table for extracting the estimated probability of requiring RBC packages during or shortly after surgery.

Score Value	Estimated Probability	Score Value	Estimated Probability
0	0.13%	7	31.13%
1	0.29%	8	51.16%
2	0.67%	9	70.82%
3	1.54%	10	84.91%
4	3.5%	11	92.88%
5	7.76%	12	96.8%
6	16.32%	13	98.59%

**Table 6:** Examples for using the scoring system. For patient 1, a lumbar spine surgery (+1) with a vertebral body replacement (+1) and 0 stages (+0) is planned. The pre-operative Hb is 9.1 g/dL (+5). The total score for patient 1 is 1 + 1 + 0 + 5 = 7, corresponding to an estimated probability of RBC package transfusion of 31.13%. For patient 2, a combined surgery (+2) with no vertebral body replacement (+0) and more than 3 stages (+3) is planned. The pre-operative Hb is 11.7 g/dL (+5). The total score for patient 2 is 2 + 0 + 3 + 5 = 10, corresponding to an estimated probability of RBC package transfusion of 84.91%.

Patient	Risk Factor	Type of Surgery	Vetebral Body Replacement	Stages	Hb (g/dL)	Total Score Value	Estimated Probability
1	Risk category	lumbar spine	yes	0	9.1	7	31.13%
	Risk points	+1	+1	+0	+5		
2	Risk category	combination	no	>3	11.7	10	84.91%
	Risk points	+2	+0	+3	+5		

## Data Availability

The data presented in this study are available on request from the corresponding author.

## Supplementary Materials

The following supporting information can be downloaded at: https://www.mdpi.com/article/10.3390/jcm13040948/s1, Example code.
